# From biological association to service integration: a public administration perspective on oral microbiota and chronic kidney disease

**DOI:** 10.1080/20002297.2026.2704279

**Published:** 2026-07-30

**Authors:** Yuyun Eka Kartika Sari, Deby Febriyan Eprilianto, Ahmad Yusroni, Muhammad Khairul Anwar

**Affiliations:** a Applied Bachelor Program in Public Administration, Faculty of Vocational Studies, Universitas Negeri Surabaya, Surabaya, Indonesia; b Public Administration, Faculty of Social and Political Sciences, Universitas Negeri Surabaya, Surabaya, Indonesia

To the Editor,

We read with great interest the review by Fang et al., ‘Relationship between oral microbiota and chronic kidney disease: facts and perspectives’ [1]. The article offers a valuable synthesis of the possible biological pathways linking oral dysbiosis, periodontitis, systemic inflammation, endothelial dysfunction, oxidative stress, and renal disease progression. Its careful statement that causality remains unproven is particularly important, because the oral-kidney axis is clinically promising but still vulnerable to overinterpretation.

We would like to add a complementary perspective from public administration and health service governance. The relationship between oral microbiota and chronic kidney disease (CKD) should not be viewed only as a biological question. It is also a service-delivery question. In daily health systems, oral care and renal care are often organised as separate services, managed through different referral pathways, financing arrangements, professional cultures, and information systems. This fragmentation may influence who receives preventive dental assessment, who is referred early, and whose periodontal condition is documented as part of CKD management.

This point matters for future oral microbiome research. If patients with CKD have different access to dental services, health insurance coverage, health literacy, medication management, or referral support, then microbial findings may partly reflect the organisation of care rather than biology alone. In other words, oral dysbiosis may be shaped not only by host-microbe interaction, but also by the administrative environment in which prevention, screening, and treatment are delivered. Variables such as dental visit history, affordability of periodontal treatment, referral from nephrology clinics, availability of integrated electronic records, and continuity of care should therefore be treated as meaningful contextual data, not merely background information.

Fang et al. rightly note that current interventional evidence is limited by small samples, short follow-up, heterogeneous periodontal interventions, inconsistent renal endpoints, limited blinding, and inadequate adjustment for confounders [1]. We suggest that future studies could extend this concern by including a minimum ‘service integration dataset.’ Such a dataset might include whether CKD patients had routine oral screening, whether dental and renal services shared referral mechanisms, whether periodontal treatment was covered or paid out-of-pocket, whether patients received oral health education from renal care providers, and whether follow-up was feasible within the local health system. These variables would not weaken microbiological analysis. Instead, they would make findings more interpretable and more useful for implementation.

This perspective is aligned with the global shift toward integrating oral health into universal health coverage. The World Health Organisation has emphasised that oral health remains a major public health concern and that reform requires policy action across health systems [2]. For CKD patients, especially those with multimorbidity or limited resources, the issue is not simply whether periodontal treatment is biologically beneficial, but whether the service system allows them to receive it consistently, affordably, and early enough to matter.

We therefore encourage future oral microbiota-CKD studies to combine mechanistic rigour with implementation sensitivity. Randomised trials and cohort studies should continue to refine microbial and renal endpoints, but they should also document the policy and service conditions under which interventions occur. This would help distinguish between three possibilities: oral dysbiosis as a biological driver of renal deterioration, oral dysbiosis as a marker of broader health vulnerability, or oral dysbiosis as a partially modifiable outcome of fragmented preventive care.

The review by Fang et al. makes an important contribution by clarifying the biological plausibility of the oral-kidney relationship. Adding a public administration lens may help move the field from association toward action: from identifying microbial patterns to designing integrated, equitable, and feasible oral health strategies for people living with CKD.
